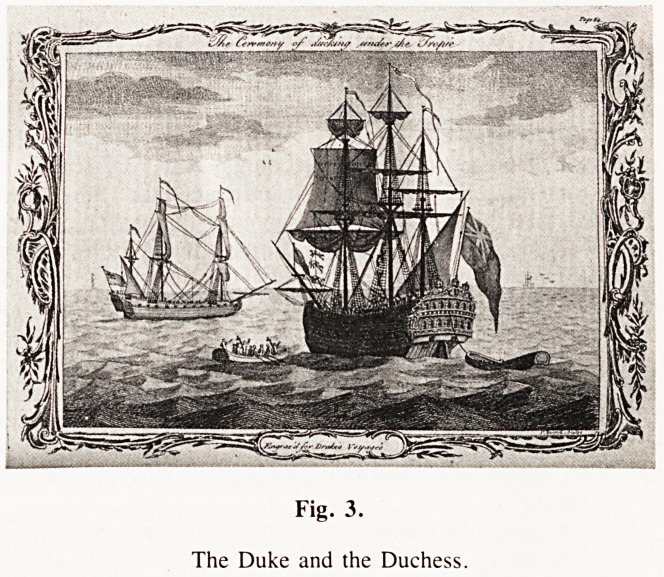# Captain Thomas Dover

**Published:** 1991-12

**Authors:** John Crossley


### Captain Thomas Dover, His background and early years

**John Crossley, MB, BChir, FRCS, FRCOG**

Thomas Dover was born in 1662, two years after the Restoration, at Barton on the Heath in a house still standing. His grandfather Robert read law at Gray's Inn, practised at Barton on the Heath and achieved local popularity and fame as the originator of the Cotswold Games which still takes place on Dover Hill in the parish of Weston sub Edge. The games, known as the British Olympics continued until 1852 when they were disbanded because of hooliganism. They have been restarted in this century and take place on the first weekend in May. The games consisted of various contests at single stick (backsword), wrestling, running, jingling, Morris dancing, greyhound coursing and horse racing. It is said that Shakespeare, whose aunt Joan Arden lived at Barton on the Heath, attended the games and included scenes from and about them in several plays, especially the wrestling scene in *As you like it*. Justice Shallow in *The Merry Wives of Windsor* asks "How does your fellow greyhound Sir? I heard say he was outrun at Cotteswold", Shakespeare's familiarity with legal terms may have resulted from his association with Dover.

The Games were celebrated in a book *Annalia Dubrensia* written by 33 Poets In 1636, amongst whom were Ben Johnson and Michael Drayton. On the cover of the book is a delightful picture depicting various activities of the games and showing Dover giving the signal to start the games, mounted on a white horse and dressed in a suit of the King's (James 1st) cast off clothing, obtained for him by Endymion Porter, a friend of Dover and a servant of the King. A yellow flag was run up on a flagpole on a temporarily erected Dover Castle and two cannon, mounted in the towers were let off.

Robert's son John, one of four children, married Elizabeth Bade, related to the Traceys of Stanway Hall. The Tracey family became lifelong friends of Thomas and he spent his last months at Stanway Hall and is buried in the Tracey family vault. John Dover became Captain of Horse under Prince Rupert and after the King's defeat retired to farm his land at Barton. Of John's 3 sons only two achieved adult life. John went to Magdalen College, read law at Grey's Inn, became a playwright (not a very good one) then took holy orders and became Rector of Drayton. His father thought little of him and virtually disinherited him.

Thomas, his favourite son was reared as a sportsman, taught to ride, handle a sword and shoot with musket and pistol. Skills which no doubt contributed to success in his later career as privateer and as a ship's captain in charge of mariners. One of Thomas's three sisters married Samuel Hopkins, an apothecary who later accompanied Dover on the voyage round the world.

Thomas was educated at Chipping Camden Grammar school and Magdalen Hall, Oxford which was a Grammar School connected to Magdalen College. He graduated Bachelor of Arts in 1684 and then moved to Gonville and Caius College at Cambridge and graduated Bachelor of Medicine in 1687. He then moved to London where he became a house pupil of the great Dr. Sydenham who resided in the Mall next door to his apothercary Dr. Malthus, great grandfather of the Reverend Dr. Malthus the philosopher famous for his concern about over population. Sydenham's great contribution to medicine was the clinical study of disease by detailed observation. In 1787, by the time Dover was his pupil he was suffering seriously from gout and died in 1689. His personal character has been universally recognised as being noble modest and sincere. His observations on epidemic diseases have been the model of many similar researches. He gave the first clear description of chorea, hysteria, and several other diseases and introduced the 'cooling treatment' for smallpox.

Dover returned to practice and to look after his estate at Barton Hill. He married in 1687 and had four daughters two of whom died in childhood. Elizabeth, one of the survivors married a John Opie, who later became a ship's officer in the East India Company and helped Dover and Woodes Rogers bring back to England the prizes they had captured on their privateering expedition.

In 1696 Dover moved to Bristol, settled in a house in the newly built Queen Square and was the first doctor to offer his services free to St. Peter's Hospital. Formerly the Mint, the building was one of the first workhouse hospitals in the country. Dover also built up a big and fashionable practice amongst the rich merchants. His day would consist of a visit to the Hospital, and then to the West India Coffee House near the Corn Exchange where he would meet his apothecary. The apothecaries, the forerunners of the general practitioners, dispensed most of the medicines and frequently only discussed patients with the physicians and often called them in too late. Many physicians, Dover among them, objected to the stranglehold the apothercaries had. Some of them started to dispense their own medicines and this division amongst the Fellows of the Royal College of Physicians resulted in the Dispensarians and the Non-dispensarians. Knowledge of disease was then rudimentary but many herbal medicines then dispensed had a sound basis e.g. cinchona bark for malaria. Small beer was used widely to replace fluids in fevers. The use of opium for pain was stressed by Sydenham and Dover perpetuated his master's teaching in the powder which bears his name. Ipecacuana was added to make the patients vomit if they attempted to take too large a dose.

**Fig. 1.**

The Cotswold Games

IPECACUANHA AND OPIUM POWDER
(Pulvis Ipecacuanhae et Opii)

*Synonyms:* Dover's Powder; Pulvis Ipecacuanhae Compositus

| | |
|---|---|
| Lactose, finely powdered | 800 g |
| Prepared Ipecacuanha | 100 g |
| Powdered Opium | 100 g |

Mix, as described under Powders.

**Standard**

**Presence of ipecacuanha and of opium.** It exhibits the microscopical characters described under Powdered Ipecacuanha and under Powdered Opium given in the British Pharmacopoeia.

**Content of anhydrous morphine.** 0·90 to 1·10 per cent, determined by the following method:

Triturate about 5 g, accurately weighed, for 3 minutes in a porcelain dish with a mixture of 3 ml of *alcohol (95 per cent)* and 1 ml of *dilute ammonia solution*, and add gradually, with stirring, sufficient *anhydrous alumina* (about 10 to 15 g) to produce a free-flowing powder; transfer the powder to a dry chromatography tube, previously lightly plugged with cotton wool immediately above the tap;

**Fig. 2.**

Dover's Powder.

There next follows a period of a few years unaccounted for in Dover's life. It seems likely that it was spent either as a ship's captain or in some way connected with the slave trade because in 6 years Dover was able to invest £3,000 pounds in a privateering voyage.

In association with Woodes Rogers, a wealthy Bristol merchant and a potter who had a factory at the bottom of Jacob's Wells road, he obtained enough backing to fit out two ships, the *Duke* and the *Duchess* and to embark on a voyage which for many reasons will ensure that his name is never forgotten.

**Fig. 3.**

The Duke and the Duchess.

## Figures and Tables

**Fig. 1. f1:**
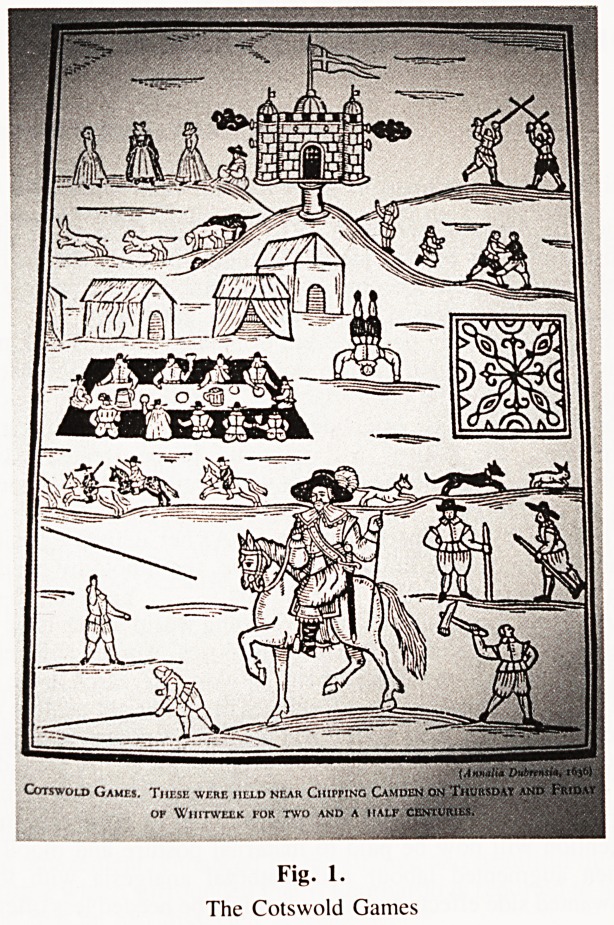


**Fig. 2. f2:**
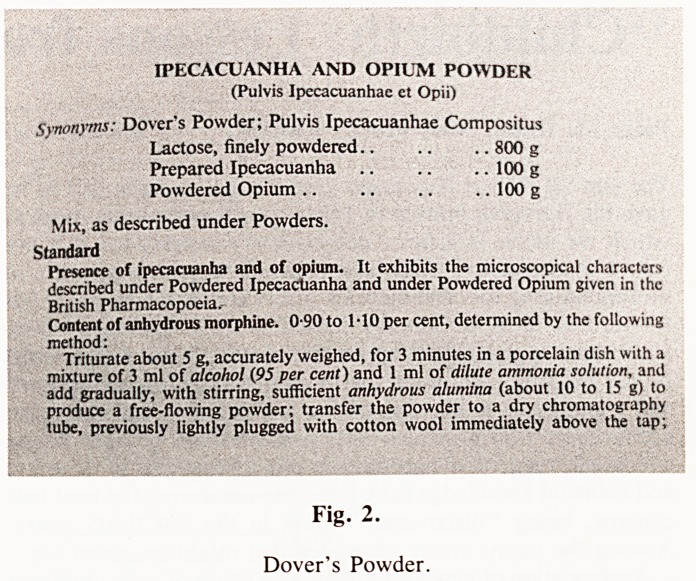


**Fig. 3. f3:**